# Unreacted RSV Polymerase Structures Expand the Post-Translocation Landscape of the Nucleotide Addition Cycle

**DOI:** 10.3390/microorganisms14081801

**Published:** 2026-08-15

**Authors:** Dongdong Cao, Eleanore Zhou, Jun Sim, Bo Liang

**Affiliations:** Department of Biochemistry, Emory University School of Medicine, Atlanta, GA 30322, USA; dongdong.cao@emory.edu (D.C.);

**Keywords:** respiratory syncytial virus (RSV), RNA-dependent RNA polymerase, nucleotide addition cycle, RNA elongation, post-translocation intermediate, conformational dynamics, cryo-electron microscopy (cryo-EM)

## Abstract

Respiratory syncytial virus (RSV) RNA synthesis relies on RNA-dependent RNA polymerase, a complex composed of the L and P proteins. We recently resolved four major structural states of the RSV nucleotide addition cycle (NAC), establishing a framework for viral RNA elongation. However, reaction-derived complexes are influenced by catalytic efficiency and intermediate occupancy and may incompletely sample the NAC conformational landscape. Here, we report two cryo-electron microscopy (cryo-EM) structures of unreacted RSV polymerase bound to a post-translocation RNA product mimic. One structure closely recapitulates the previously determined reaction-derived post-translocation state, demonstrating that the canonical architecture can be recovered independently of nucleotide incorporation. The second structure reveals an additional post-translocation intermediate that retains the same RNA register and vacant +1 product-binding site while adopting a two-domain L organization. Structural comparisons further identify localized rearrangements near the catalytic center, including changes in the motif A loop and supporting helix. Together, these findings expand the structural landscape of the RSV post-translocation stage and refine the current NAC model. More broadly, our results highlight unreacted state-matched complexes as a complementary approach to reaction-derived structures for capturing more complete structural snapshots of dynamic reaction cycles.

## 1. Introduction

Respiratory syncytial virus (RSV) infection is a leading cause of severe lower respiratory tract diseases in infants, young children, older adults, and immunocompromised individuals worldwide [1,2,3,4,5]. RSV belongs to the family *Pneumoviridae* within the order of *Mononegavirales*, also known as the non-segmented, negative-sense (NNS) RNA virus [6]. Although several antibodies and vaccines against RSV have recently been approved by the FDA, antiviral development options remain limited [7,8,9,10]. The RSV RNA-dependent RNA polymerase is therefore an important target for mechanistic investigation and antiviral developments. The polymerase complex consists of the large (L) protein and the cofactor phosphoprotein (P). The L protein contains the RNA-dependent RNA polymerase (RdRp), capping (Cap), connector (CD), methyltransferase (MT), and C-terminal (CTD) domains, and the P protein includes the N-terminal (P_NTD_), oligomerization (P_OD_), and C-terminal (P_CTD_) domains [11,12]. Together, L and P catalyze RNA replication and transcription, during which the leader (Le) or trailer complementary (TrC) regions at the 3′ end of the genome and the antigenome RNA are recognized as promoters for the initiation of RNA synthesis [13].

Cryo-electron microscopy (cryo-EM) has provided increasingly detailed views of RSV polymerase architecture and function. Structures of the apo polymerase [14,15] and the promoter-bound complexes [16], provided structural insights into L-P interactions and RNA synthesis initiation mechanisms. More recently, we determined structures representing four major nucleotide addition cycles (NAC) during early-stage RNA elongation: NTP-bound, pre-reaction, pre-translocation, and post-translocation [17]. These structures revealed coordinated changes at multiple structural scales. Two of the states, NTP-bound and pre-reaction, were captured before the catalysis of phosphodiester bond formation. However, the other two states, pre-translocation and post-translocation, were obtained after catalysis and are termed post-catalytic states. The NTP-bound and post-translocation states adopted a five-domain L architecture, whereas the pre-reaction and pre-translocation states displayed a two-domain architecture in which the CD, MT, and CTD regions were not resolved. In parallel, localized rearrangements within catalytic motifs and RNA-interacting elements accompanied nucleotide binding, active-site closure, catalysis, and RNA translocation [17]. Together, these structures established a framework for the RSV NAC and suggested that additional conformational intermediates may remain unresolved between the major states.

Capturing such intermediates remains challenging because structures obtained from active reactions are shaped by the kinetics and efficiencies of multiple sequential events. For a polymerase NAC, the population of a given complex may depend on nucleotide binding, active-site closure, phosphodiester-bond formation, pyrophosphate release, RNA translocation, and subsequent conformational rearrangements. Transient or weakly populated intermediates may therefore be underrepresented in reaction-derived structural datasets. Direct assembly of polymerases with preformed substrate-, intermediate-, or product-state mimics offers a complementary approach that bypasses selected upstream reaction steps and samples conformations compatible with a defined biochemical state. Whether such unreacted, state-matched complexes can reproduce reaction-derived RSV polymerase states while revealing additional NAC intermediates has remained unclear.

Here, we directly assembled RSV polymerase with a post-translocation RNA product mimic (Tr3-mimic; TrC12-4U-Tr3) and determined two cryo-EM structures of the resulting unreacted complexes at 3.55 and 3.48 Å resolution. One structure, termed unreacted Tr3-mimic form A, closely recapitulates the previously determined reaction-derived, post-catalytic post-translocation state, demonstrating that the corresponding architecture can be recovered independently of nucleotide incorporation. The second structure, unreacted Tr3-mimic form B, retains the same post-translocation RNA register and vacant +1 product-binding site but adopts a two-domain L architecture, revealing an additional intermediate within the post-translocation stage. These findings refine the structural model of the RSV NAC and highlight unreacted state-matched complexes as a complementary strategy for expanding structural snapshots of dynamic reaction cycles. More broadly, systematic integration of reaction-derived structures with unreacted complexes assembled using preformed substrate-, intermediate-, or product-state mimics may provide a general framework for resolving conformational intermediates in other dynamic enzymes and macromolecular machines.

## 2. Materials and Methods

### 2.1. Expression and Purification of the RSV Polymerase (L:P Complex)

The RSV polymerase was expressed and purified as follows: The codon-optimized helper plasmids of the RSV (strain A2) L and P proteins were acquired from BEI Resources (Manassas, VA, USA). Subcloning was performed to incorporate the *L* and *P* genes into the pFastBac Dual vector (Thermo Fisher Scientific, Waltham, MA, USA), which contains open reading frame 1 (ORF1) and open reading frame 2 (ORF2). ORF1 encodes the RSV *L* gene, and ORF2 encodes the RSV *P* gene. Separation by TEV protease cleavage was performed at the N-terminus of the RSV L protein, which had a 6 × His tag. Subsequently, the transformation was performed to incorporate a recombinant pFastBac Dual vector into *Escherichia coli* DH10Bac (Thermo Fisher Scientific, Waltham, MA, USA), generating bacmid DNA.

Recombinant baculoviruses were obtained by transfecting Sf21 cells (Thermo Fisher Scientific, Waltham, MA, USA) with bacmid DNA using Cellfectin II reagent (Thermo Fisher Scientific, Waltham, MA, USA). The recombinant baculoviruses present in the suspension culture were used to infect the Sf21 cells, and the cells were harvested 72 h post-infection by centrifugation at 1000× *g* for 15 min.

The harvested cells were resuspended in lysis buffer (50 mM sodium phosphate, pH 7.4, 300 mM NaCl, 6 mM MgSO_4_, 10% glycerol, 0.2% NP-40, EDTA-free protease inhibitor), after which a homogenizer was used to lyse the cells, followed by centrifugation at 16,000× *g* for 60 min to clarify the lysate. Using a Co^2+^-NTA agarose resin (GoldBio, St Louis, MO, USA), incubation of the clarified lysate was conducted. Subsequently, a wash buffer (50 mM sodium phosphate, pH 7.4, 300 mM NaCl, 6 mM MgSO_4_, 10% glycerol, 10 mM imidazole) was used for washing. The RSV L:P complexes were eluted by an elution buffer (50 mM sodium phosphate, pH 7.4, 300 mM NaCl, 6 mM MgSO_4_, 10% glycerol, 250 mM imidazole). Afterward, a TEV enzyme was added to the eluted sample, and subsequently, the sample was added to a Co^2+^-NTA agarose resin. Application of the flow-through sample to the heparin column (Cytiva, Marlborough, MA, USA) and further purification were carried out by size exclusion chromatography with gel filtration buffer (25 mM HEPES, pH 7.4, 300 mM NaCl, 6 mM MgSO_4_, 0.5 mM tris(2-carboxyethyl) phosphine hydrochloride [TCEP]) utilizing a Superose 6 Increase 10/300 GL column (GE Healthcare, Chicago, IL, USA). Pure proteins were stored at −80 °C in 30 μL aliquots and flash-frozen in liquid nitrogen for further use. These procedures have been described previously [16].

### 2.2. Cryo-EM Grid Specimen Preparation and Data Acquisition

The RNA oligo Tr3-mimic was purchased from Integrated DNA Technologies (Coralville, IA, USA) for structural analysis. Here, 0.3 mg/mL of purified RSV polymerase was incubated with 30 µM of Tr3-mimic for 1 h at 30 °C. Then, 3.0 μL of the assembled complex samples were applied to glow-discharged UltrAuFoil 300-mesh R1.2/1.3 grids (Electron Microscopy Sciences, Hatfield, PA, USA). Grids were blotted at ∼100% humidity for 3 s and, using a FEI Vitrobot Mark IV (Thermo Fisher Scientific/FEI, Hillsboro, OR, USA), were flash-frozen with liquid ethane.

The Thermo Fisher Scientific (TFS) Titan Krios electron microscope (Thermo Fisher Scientific/FEI, Hillsboro, OR, USA), equipped with a Gatan K3 camera (Gatan, Pleasanton, CA, USA) and operating at an acceleration voltage of 300 kV, was used to collect the images. A defocus range of −0.6 to −2.5 μm was set. Dose-fractionated images were recorded with a 40 ms per-frame exposure time (40 frames) and a dose of 1.2 to 1.3 electrons per Å^2^ per frame. A total of 3888 micrographs were collected for the RSV polymerase in complex with Tr3-mimic (Figure S1 and Table S1).

### 2.3. Cryo-EM Data Processing

The cryo-EM data of the RSV polymerase incubated with Tr3-mimic were processed by cryoSPARC v4.5.1. [18] After ‘Patch Motion Correction’ [19] and ‘Patch CTF refinement’, 3,558,478 particles were automatically picked from the aligned micrographs using a circular blob with a diameter ranging from 100 to 145 Å by ‘Blob Picker’, and extracted using a cropped 64-pixel box size. The extracted particles were further processed with ‘2D classification’, and 916,396 particles were selected and extracted using a 208-pixel box size (1.058 Å/pixel) for further data processing. Following ‘Ab Initio Reconstruction’ and ‘Heterogeneous Refinement’, the 3D classes with good density for the RNA were selected for further ‘Heterogeneous Refinement’. One 3D class displaying density for both template and product RNAs and the CD domain of L was selected for further ‘Heterogeneous Refinement’ followed by ‘Non-uniform Refinement’ on the 3D class with the best-resolved Tr3 density, resulting in a map of unreacted Tr3-mimic form A at 3.55 Å resolution with 26,389 particles. Separately, another 3D class displaying density for template and product RNAs but not the CD domain was selected for further ‘Heterogeneous Refinement’. Further ‘Non-uniform Refinement’ on the 3D class with the best-resolved density for Tr3 results in a map of unreacted Tr3-mimic form B at 3.48 Å resolution with 36,361 particles (Figure S1 and Table S1). All reported resolutions were based on the Fourier shell correlation (FSC)  =  0.143 criterion and gold-standard refinement procedures.

### 2.4. Model Building and Figure Preparation

The coordinates of the NTP-bound RSV polymerase (PDB: 9PFR) were used as the initial model to fit into the cryo-EM maps using UCSF ChimeraX v1.5 and Coot v0.9.8.7 [20,21]. All RSV polymerase models were built and refined using Coot v0.9.8.7 [21] and PHENIX v1.19.2-4158 [22]. MolProbity v4.5.1 was used to validate the geometries of models [23]. The data collection and model refinement statistics are shown in Table S1. Coot v0.9.8.7 [21], PyMOL v2.5.4 [24], and UCSF ChimeraX v1.5 [20] were used to generate all the electron density maps and represent the models in the figures. Together, the software for this project was curated by SBGrid (https://sbgrid.org/, accessed on 1 January 2026) [25].

## 3. Results

### 3.1. Cryo-EM Structures of Unreacted RSV Polymerase Bound to a Post-Translocation RNA Mimic

**Figure 1:**
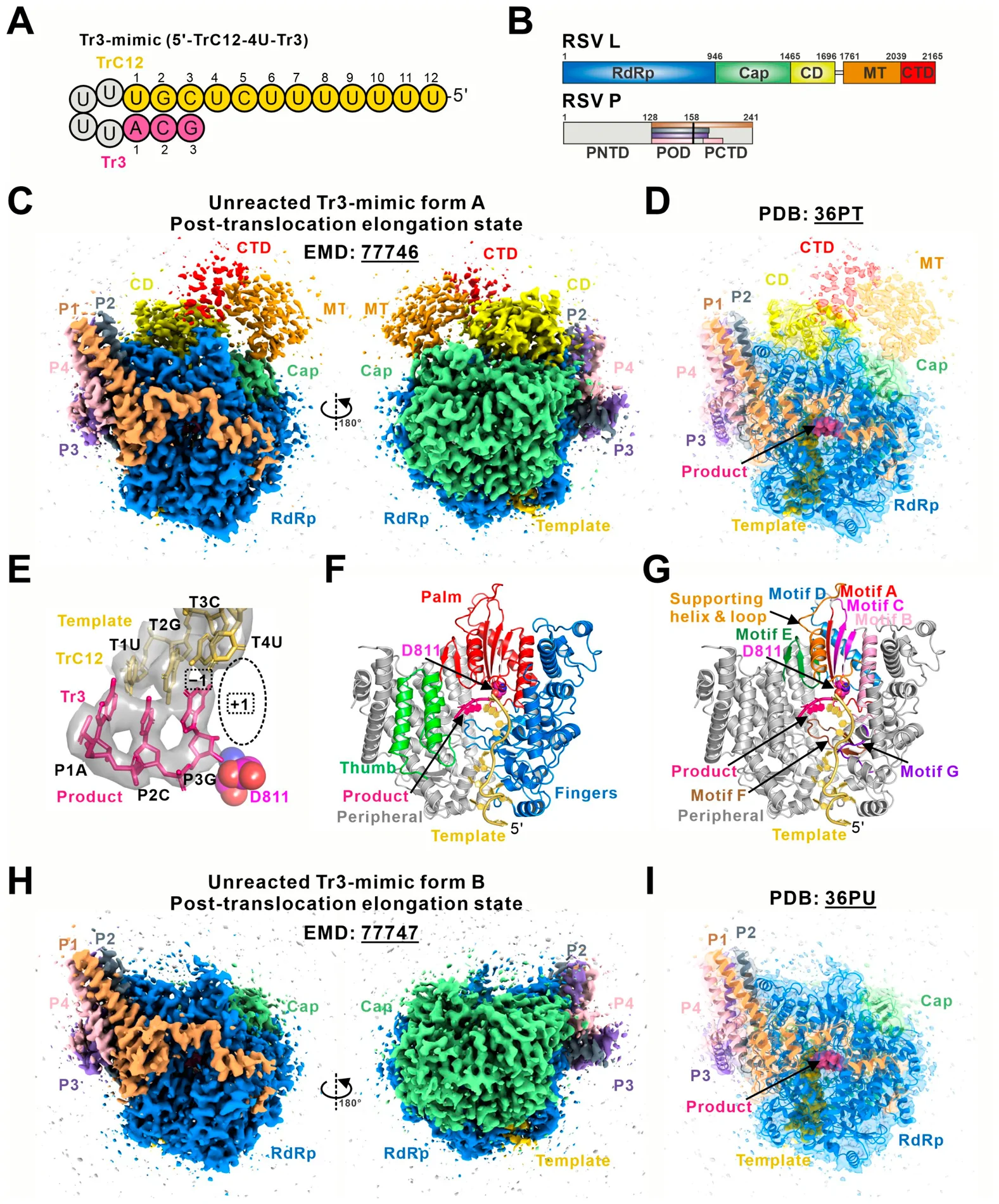
Cryo-EM structures of RSV polymerase in complex with Tr3-mimic (TrC12-4U-Tr3). (**A**) Sequence and structure of the Tr3-mimic RNA (5′-TrC12-4U-Tr3). The template TrC12 is colored yellow-orange, the primer Tr3 is colored hot pink, and the tetra-U (5′ UUUU) is colored gray. (**B**) Schematic domain organization of the RSV polymerase (L:P complex) with labeled boundaries. The L protein contains the RNA-dependent RNA polymerase (RdRp, blue), capping (Cap, green), connector (CD, yellow), methyltransferase (MT, orange), and C-terminal (CTD, red) domains, and the P protein includes the N-terminal (P_NTD_), oligomerization (P_OD_), and C-terminal (P_CTD_) domains. Four P monomers are colored bright orange (P1), gray (P2), purple-blue (P3), and pink (P4). (**C**) The cryo-EM map of the unreacted Tr3-mimic form A (EMD: 77746), contoured at 0.20. (**D**) The cartoon diagram of the unreacted Tr3-mimic form A (PDB: 36PT) fits into the cryo-EM map. The cryo-EM density for the MT and CTD domains is not sufficient for model building. (**E**) The close-up view of the RNAs of the unreacted Tr3-mimic form A fitted in the cryo-EM map. (**F**) The overview of the conventional right-hand ‘fingers-palm-thumb’ fold of the RSV L RdRp domain and RNAs in the unreacted Tr3-mimic form A. (**G**) The motifs A to G of the RSV polymerase, along with the RNA template and product, are highlighted in the unreacted Tr3-mimic form A. The catalytic residue D811 is shown as a magenta sphere. (**H**) The cryo-EM map of the RSV polymerase in unreacted Tr3-mimic form B (EMD: 77747), contoured at 0.20. (**I**) The cartoon diagram of the RSV polymerase in unreacted Tr3-mimic form B (PDB: 36PU) fits into the cryo-EM map. The PDB and EMD IDs are underlined.

In our previous study, we designed hairpin-loop RNA duplexes to investigate the mechanism of RSV RNA synthesis during RNA elongation [17]. These constructs contain an RNA template derived from the 3′-terminal 12 nucleotides (nts) of the trailer complementary promoter (TrC12, 3′-UGCUCUUUUUUU) and an RNA primer (Tr2, 5′-AC or Tr3, 5′-ACG), connected by a tetra U loop, termed Tr2-mimic (TrC12-4U-Tr2) or Tr3-mimic (TrC12-4U-Tr3) (Figure 1A). Using these hairpin-loop RNA duplexes, we captured snapshots of RSV polymerase in four nucleotide-addition cycle (NAC) states: NTP-bound (9PFR), pre-reaction (9PFS), pre-translocation (9PFT), and post-translocation (9PFU). The NTP-bound and post-translocation states display a five-domain L architecture, whereas the pre-reaction and pre-translocation states show a two-domain (RdRp and Cap) L conformation with no density observed for the CD, MT, and CTD domains of L.

The previously determined post-translocation state (post-catalytic Tr3-mimic) was captured by incubating the RSV polymerase with Tr2-mimic and GTP. Following GTP incorporation, the resulting Tr3 product is translocated by one nucleotide, vacating the +1 position at the product site and yielding a reaction-derived, post-catalytic post-translocation complex. However, the population of this complex was constrained by the efficiency of nucleotide incorporation and subsequent translocation under the in vitro reaction conditions. To directly sample polymerase conformations compatible with the post-translocation RNA register, we instead incubated RSV polymerase with the preformed Tr3-mimic product (Figure 1A). Cryo-EM analysis of this unreacted complex yielded two distinct reconstructions, termed unreacted Tr3-mimic forms A and B, at overall resolutions of 3.55 and 3.48 Å, respectively (Figure S1 and Table S1).

The unreacted Tr3-mimic form A (PDB: 36PT; EMD-77746) displays densities for the oligomerization (P_OD_) and C-terminal domains (P_CTD_) of the tetrameric P as well as the five domains (RdRp, Cap, CD, MT, and CTD) of L, representing the five-domain conformation (Figure 1B,C). The density quality of the MT and CTD domains of L is poor, with only fragments. Based on the cryo-EM map, we built models of the L RdRp, Cap, and CD domains, as well as the P_OD_ and P_CTD_ of P (Figure 1D). In this model, the first 4 nts at the 3′ end of the template (T1U-T4U) sit in the catalytic pocket, with T1U-T3C pairing with the Tr3 (5′-ACG) product chain (Figure 1E and Figure S2A). The +1 position on the product site is empty, representing a post-translocation state. The rest of the template is located in the template entrance channel. The Tr3-mimic RNA is closely interacting with the L RdRp domain, which adopts a conventional ‘fingers-palm-thumb’ right-hand polymerase fold (Figure 1F). Seven catalytic motifs A–G, as parts of the catalytic pocket, coordinate the RNA synthesis (Figure 1G).

The same dataset also yielded unreacted Tr3-mimic form B (PDB: 36PU; EMD-77747), which adopts a two-domain L conformation of RSV polymerase, containing the RdRp and Cap domains of L and the P_OD_ and P_CTD_ of P, with no density observed for the CD, MT, and CTD domains of L as well as the P_NTD_ of P (Figure 1H and Figure S1). Despite this difference in global polymerase organization, the Tr3-mimic occupies the same post-translocation RNA register observed in form A, with the +1 position at the product site remaining vacant (Figure 1I and Figure S2B). Thus, direct assembly with the preformed Tr3-mimic captured two structurally distinct RSV polymerase complexes associated with the same post-translocation RNA register.

### 3.2. Unreacted Tr3-Mimic Form A Recapitulates the Reaction-Derived Post-Translocation State

**Figure 2:**
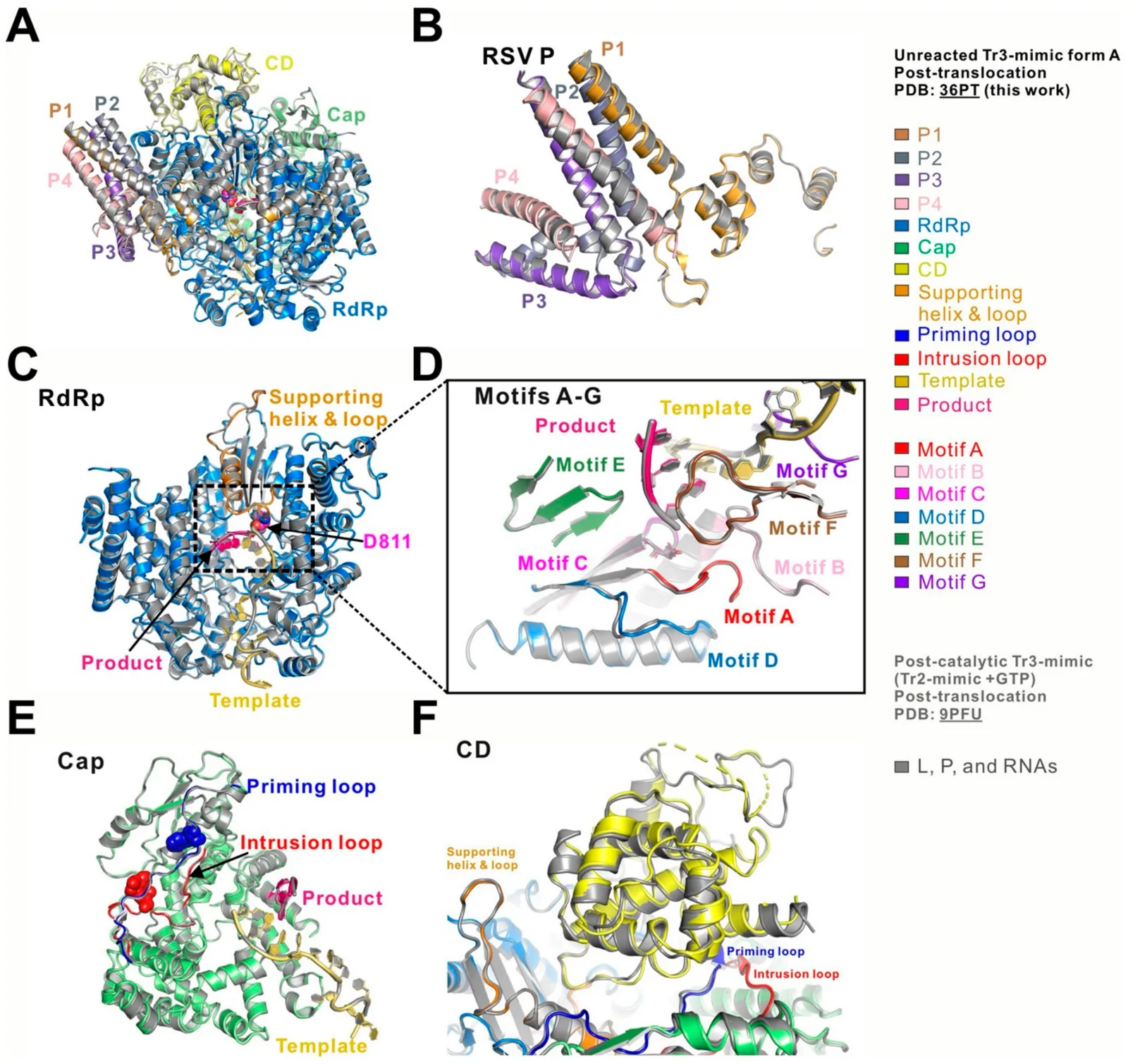
Unreacted Tr3-mimic form A recapitulates the reaction-derived post-translocation state. (**A**) The overall structural comparison between the unreacted Tr3-mimic form A (this work, PDB: 36PT) and the reaction-derived post-catalytic Tr3-mimic (Tr2-mimic + GTP, PDB: 9PFU). (**B**) The structural comparison of the RSV P proteins in two structures. (**C**) The structural comparison of the RdRp domains in two structures. (**D**) The structural comparison of the catalytic motifs A–G in two structures. (**E**) The structural comparison of the Cap domains in two structures. The priming loop in the Cap domain of the unreacted Tr3-mimic form A is colored blue, and the intrusion loop is colored red. (**F**) The structural comparison of the CD domains in two structures. The color of each domain and motif in the unreacted Tr3-mimic form A (PDB: 36PT) is indicated in the right part of the figure. The post-catalytic Tr3-mimic (PDB: 9PFU) is shown in gray. The colors of motifs A to G apply only to panel D. The PDB ID is underlined.

The unreacted Tr3-mimic form A was obtained by directly incubating RSV polymerase with the preformed Tr3-mimic product, whereas the previously determined post-translocation complex was generated through GTP incorporation into the Tr2-mimic, followed by RNA translocation [17]. Despite these distinct routes of complex assembly, the two structures adopt nearly identical overall architectures (Figure 2A). Superposition of unreacted Tr3-mimic form A (PDB: 36PT) with the reaction-derived, post-catalytic Tr3-mimic complex (PDB: 9PFU) yielded a root-mean-square deviation (RMSD) of 0.371 Å across 1683 aligned Cα atoms. Both complexes display the same overall organization of the L and P proteins, with the resolved regions of the tetrameric P cofactor adopting closely similar conformations (Figure 2A,B).

Domain-level comparisons further demonstrate the close structural agreement between the two complexes. The RdRp domains superimpose closely, including the supporting helix and associated loops (Figure 2C), and the conserved catalytic motifs A–G adopt equivalent conformations around the active-site cavity (Figure 2D). The Cap domains are similarly aligned, with the priming and intrusion loops occupying comparable positions (Figure 2E). The resolved portions of the CD domains also exhibit closely similar architectures (Figure 2F). Minor differences are primarily confined to regions with weaker cryo-EM density, including several loops in the CD domain of unreacted Tr3-mimic form A that could not be modeled reliably. In both complexes, the density for the MT and CTD domains is insufficient to build complete atomic models.

Thus, direct assembly of RSV polymerase with a preformed post-translocation RNA product mimic recapitulates the architecture of the corresponding reaction-derived post-translocation complex. This structural agreement indicates that the five-domain post-translocation architecture is independent of the experimental route used to generate the Tr3 product and can be accessed without nucleotide incorporation or translocation under the assembly conditions used here.

### 3.3. Unreacted Tr3-Mimic Form B Reveals an Additional Post-Translocation Intermediate

**Figure 3:**
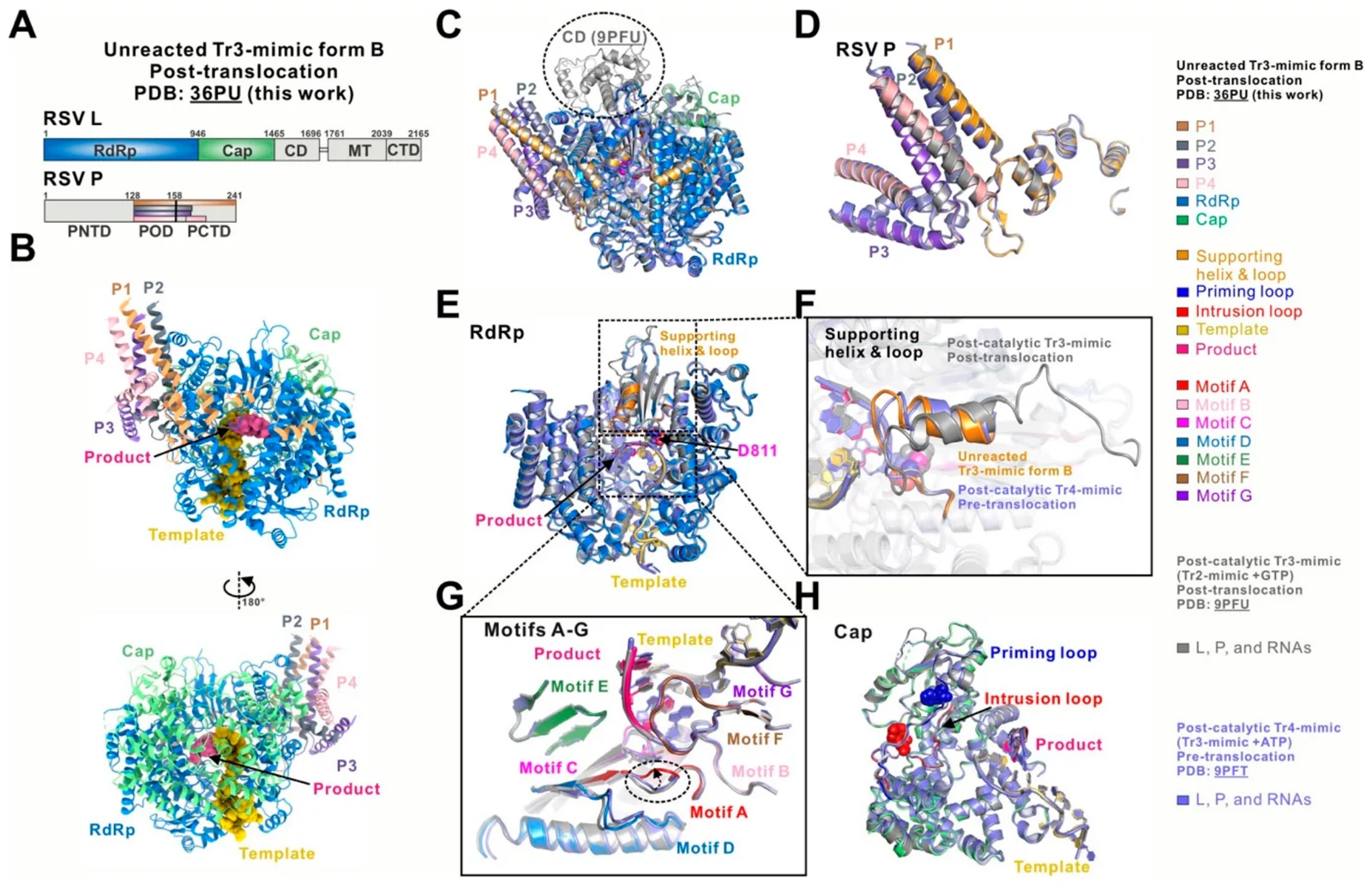
Unreacted Tr3-mimic form B (this work, PDB: 36PU) reveals an additional two-domain post-translocation intermediate. (**A**) Schematic domain organization of the RSV polymerase (L:P complex) in unreacted Tr3-mimic form B with labeled boundaries. There is no cryo-EM density observed for the CD, MT, and CTD domains of the L protein. (**B**) The cartoon diagrams of the RSV polymerase in the unreacted Tr3-mimic form B (this work, PDB: 36PU). (**C**) The overall structural comparison among the unreacted Tr3-mimic form B (this work, PDB: 36PU), the post-catalytic Tr3-mimic (post-translocation state, PDB: 9PFU, gray), and the post-catalytic Tr4-mimic (pre-translocation state, PDB: 9PFT, light blue). (**D**) The structural comparison of the RSV P proteins in these three structures. (**E**) The structural comparison of the RdRp domains in these three structures. (**F**) The structural comparison of the supporting helix and loops in the RdRp domains of these three structures. (**G**) The structural comparison of the catalytic motifs A–G in these three structures. (**H**) The structural comparison of the Cap domains in these three structures. The priming loop in the Cap domain of the unreacted Tr3-mimic form B is colored blue, and the intrusion loop is colored red. In the panels (**C**–**H**), the color of each domain or motif in the unreacted Tr3-mimic form B (PDB: 36PU) is indicated in the right part of the figure. The colors of motifs A to G apply only to panel G. The post-catalytic Tr3-mimic (PDB: 9PFU) is colored gray, and the post-catalytic Tr4-mimic (PDB: 9PFT) is colored light blue in panels (**C**–**H**). The PDB ID is underlined.

The same cryo-EM dataset also yielded unreacted Tr3-mimic form B (PDB: 36PU), which adopts a two-domain L architecture distinct from the five-domain organization of form A (Figure S1). In form B, the RdRp and Cap domains of L and the P_OD_ and P_CTD_ regions of P are resolved, whereas no interpretable density is observed for the CD, MT, and CTD domains of L or the P_NTD_ region of P (Figure 3A,B). The atomic model contains 10 nucleotides of the TrC12 template and all 3 nucleotides of the Tr3 product (Figure S2B). Importantly, the RNA retains the post-translocation register observed in form A and in the previously determined reaction-derived post-translocation complex, with the +1 position at the product site remaining vacant. Thus, form B combines a post-translocation RNA configuration with a two-domain L architecture.

To define the structural features of form B within the RSV NAC, we compared it with the previously determined reaction-derived pre-translocation complex (post-catalytic Tr4-mimic, PDB: 9PFT) and post-translocation complex (post-catalytic Tr3-mimic, PDB: 9PFU) (Figure 3C). Form B and the pre-translocation complex both display two-domain L architectures, whereas the canonical post-translocation complex contains the additional resolved CD domain within an overall five-domain organization (Figure 3C). The resolved regions of the P proteins remain closely similar among the three complexes (Figure 3D), and the overall RdRp domains also superimpose closely (Figure 3E). These comparisons indicate that the major structural distinction is concentrated in the organization and local ordering of selected L-protein elements rather than in a global rearrangement of the conserved RdRp core.

A prominent difference involves the supporting helix and associated loops of the RdRp domain (Figure 3F). In form B and the pre-translocation complex, only part of this region can be modeled, whereas in the canonical post-translocation complex, the supporting helix and associated loops are more fully resolved and contribute to interactions between the RdRp and CD domains. The reduced ordering of this region in form B is therefore associated with the absence of a resolved CD domain and distinguishes this complex from the canonical five-domain post-translocation architecture.

Additional differences are observed near the catalytic center. In form B, the motif A loop shifts inward toward the Tr3 product and the motif C region, whereas the corresponding loop adopts a more outward position in the previously determined pre-translocation and canonical post-translocation complexes (Figure 3G). This localized rearrangement identifies a distinct active-site configuration within the post-translocation RNA register. By contrast, the Cap domains remain closely similar among the three structures, with both the priming and intrusion loops retracted from the catalytic center (Figure 3H).

Together, these structural features identify unreacted Tr3-mimic form B as an additional intermediate within the post-translocation stage of the RSV NAC. Form B retains the characteristic post-translocation RNA register while combining a two-domain L architecture with distinct local organization of the supporting helix region and motif A loop. These observations further indicate that progression through the post-translocation stage involves conformational changes at both global and local structural scales.

## 4. Discussion

RSV belongs to the family of *Pneumoviridae* in the order of *Mononegavirales*, which also includes other highly pathogenic agents, such as rabies virus (RABV), Measles virus (MeV), Nipah virus (NiV), and Ebola virus (EBOV). The development of cryo-EM contributes to the determination of the high-resolution structures of the RNA polymerases from NNS RNA viruses, including RSV [14,15,16,17] and human metapneumovirus (HMPV) [26] from the *Pneumoviridae* family; vesicular stomatitis virus (VSV) [27,28] and RABV [29] from the *Rhabdoviridae* family; EBOV [30,31,32] and Marburg virus (MARV) [30] from the *Filoviridae* family; Borna disease virus (BDV) [33,34,35] from the *Bornaviridae* family, and NiV [36,37,38,39,40,41,42,43], parainfluenza virus (HPIV) [44,45], Mumps virus (MuV) [46], MeV [47], and Newcastle disease virus (NDV) [48] from the *Paramyxoviridae* family. These RNA polymerases all contain an L protein with five domains that catalyze the RNA synthesis. Interestingly, the apo-form structures of the RNA polymerase from the VSV, RABV, BDV, MuV, NDV, PIV, and MeV all feature a five-domain L conformation, whereas the apo-form polymerase structures from RSV, HMPV, NiV, EBOV, and MABV adopt a two-domain L architecture. The promoter-bound RNA polymerases of RSV and EBOV also display a two-domain L conformation. In contrast, the RNA polymerases in the NTP-bound elongation state of RSV and NiV transit into the five-domain L conformation. These observations raise a broader question: do the architectural differences among NNS viral polymerases reflect virus-specific organization, biochemical-state dependence, or incomplete sampling of a shared conformational landscape? Because most viral polymerases have been characterized in only a limited number of biochemical states, the extent and functional significance of these transitions remain unresolved.

**Figure 4:**
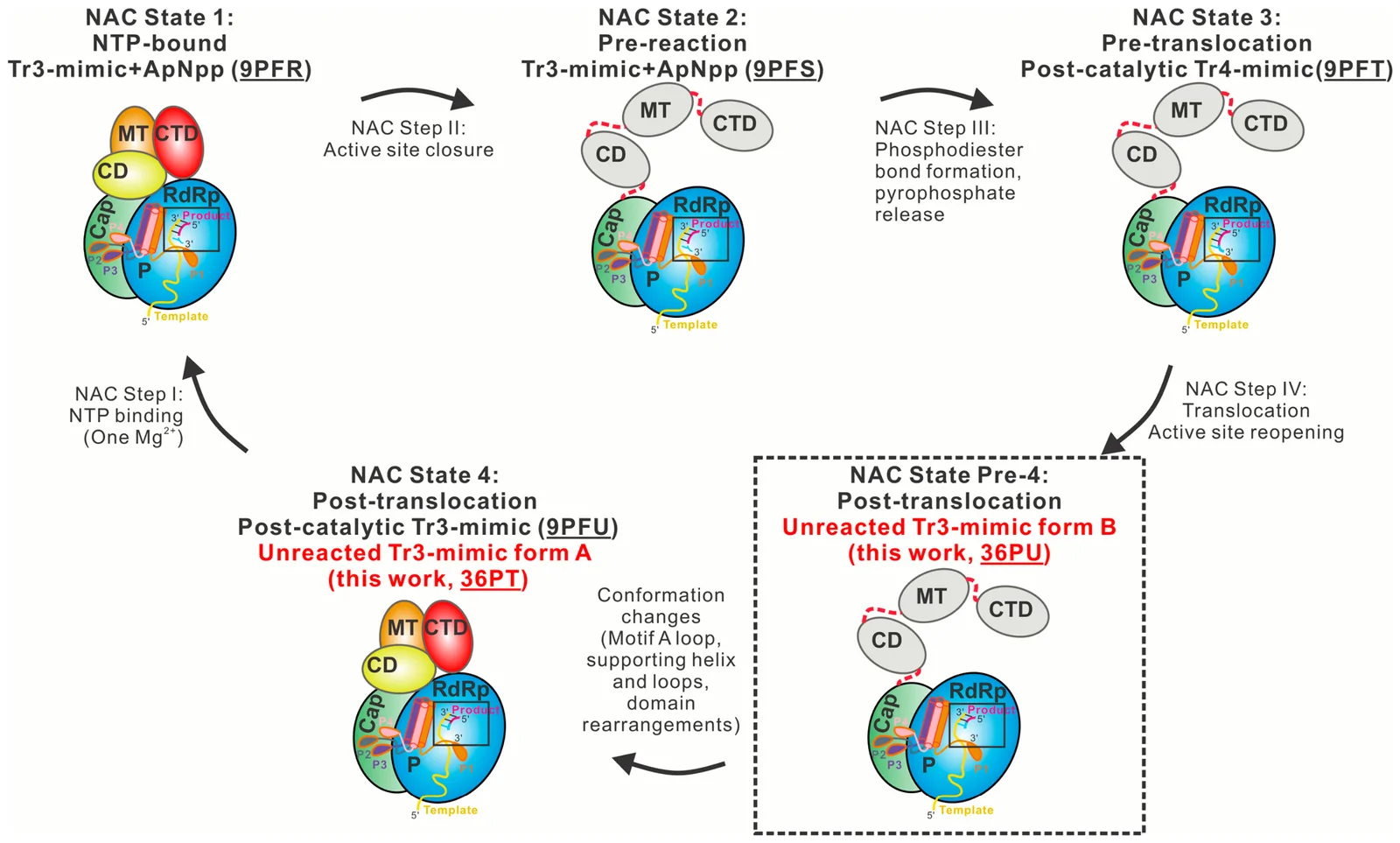
Revised structural model of the RSV nucleotide addition cycle incorporating an additional post-translocation intermediate. The NAC of the RSV polymerase involves steps (Steps I–IV) and four states (States 1–4). The NAC starts with the binding of an incoming NTP and a Mg^2+^ ion (Step I), resulting in the NTP-bound state (State 1). Secondly, the active site closure step (Step II) generates the pre-reaction state (State 2). Thirdly, the formation of the phosphodiester bond and release of the byproduct pyrophosphate (Step III) produce the pre-translocation state (State 3). Finally, the translocation step (Step IV) shifts the RNA one base pair forward to the post-translocation state (State 4), during which the active site reverts to an open conformation to bind the next incoming NTP. In this study, we report two unreacted Tr3-mimic forms of the RSV polymerase complex. The unreacted Tr3-mimic form A is the same as the previous post-translocation state (post-catalytic Tr3-mimic) structure (State 4), in which we can observe the density for all five domains of the L protein. However, the structure of the other one, the unreacted Tr3-mimic form B, shows a two-domain L structure in the complex, representing another NAC post-translocation state (State Pre-4). Note: RNA templates are shown in yellow-orange, RNA products in hot pink, and incorporated NTPs in cyan. The RSV polymerase domains are colored the same as in Figure 1B. Flexible domains are colored gray. The PDB ID is underlined.

Our previous structural analysis of the RSV nucleotide addition cycle (NAC) provided a framework for examining this question within a single viral polymerase system [17]. We identified four key NAC states during early-stage RNA elongation: NTP-bound, pre-reaction, pre-translocation, and post-translocation. Briefly, the binding of NTP (Figure 4, NAC State 1: NTP-bound) triggers active-site closure (Figure 4, NAC State 2: pre-reaction), followed by the catalysis of phosphodiester bond formation and pyrophosphate release (Figure 4, NAC State 3: pre-translocation). Translocation of the RNA moves one nucleotide forward, resulting in an empty +1 position on the product site and reopening the active site for the next NTP (Figure 4, NAC State 4: post-translocation). These structures revealed coordinated transitions at both global and local scales. The NTP-bound and post-translocation states displayed five-domain L architectures, whereas the pre-reaction and pre-translocation states adopted two-domain organizations. At the same time, localized rearrangements within catalytic motifs and RNA-interacting elements accompanied NTP binding, active-site closure, phosphodiester-bond formation, and RNA translocation. The transition between these major states is unlikely to be structurally instantaneous and may involve additional conformational intermediates that are difficult to resolve from reaction-derived datasets alone.

A central challenge in structural studies of dynamic reaction cycles is that the observed conformational ensemble depends on how a biochemical state is populated. Reaction-derived complexes are shaped by sequential events, including substrate binding, catalysis, product formation, byproduct release, translocation, and conformational rearrangements. Consequently, short-lived or weakly populated intermediates may be underrepresented in structural datasets. Direct assembly with preformed substrate-, intermediate-, or product-state mimics offers a complementary strategy by bypassing selected upstream steps and sampling conformations compatible with a defined biochemical state. Such unreacted complexes should not automatically be considered obligatory on-pathway intermediates, but comparison with reaction-derived structures can reveal reproducible state-associated architectures and additional accessible conformations.

The two unreacted Tr3-mimic structures reported here illustrate these complementary outcomes. Form A adopts a five-domain architecture that closely recapitulates the previously determined reaction-derived, post-catalytic post-translocation complex. Whereas the previous complex was generated through GTP incorporation into Tr2-mimic followed by RNA translocation, form A was assembled directly with the preformed Tr3-mimic product. Their close structural agreement demonstrates that the canonical post-translocation architecture can be accessed independently of nucleotide incorporation under the conditions examined here and provides internal validation of the state-matched assembly strategy.

Form B expands the structural landscape of the post-translocation stage. It retains the characteristic post-translocation RNA register, including a vacant +1 position at the product site, yet adopts a two-domain L architecture rather than the five-domain organization observed in form A and the canonical reaction-derived post-translocation complex. Its reduced ordering of the supporting helix region resembles features of the previously determined two-domain pre-reaction and pre-translocation states, whereas the motif A loop adopts a distinct inward position toward the Tr3 product and motif C region (Figure 3 and Figure S3). Thus, form B combines a post-translocation RNA configuration with a distinct arrangement of global and local polymerase elements, supporting its assignment as an additional intermediate within the post-translocation stage (Figure 4, NAC State Pre-4: post-translocation).

These findings emphasize that biochemical-state assignment and conformational-state assignment are not necessarily equivalent. A complex classified as post-translocation based on RNA register and active-site occupancy can encompass multiple protein conformations. More generally, a defined substrate-, intermediate-, or product-associated biochemical state may correspond to an ensemble of structural intermediates rather than a single structural endpoint. This distinction is particularly relevant to cryo-EM studies of dynamic systems, in which particle classification can resolve coexisting conformations within the same biochemical preparation. Mechanistic models based on a limited number of static structures may therefore underestimate the conformational heterogeneity within individual biochemical stages.

More broadly, reaction-derived structures and unreacted-state-matched complexes can serve as complementary approaches to reconstructing dynamic reaction landscapes. Reaction-derived trapping accesses states populated during active turnover but is constrained by reaction efficiency, intermediate lifetime, and kinetic occupancy. Unreacted assemblies with preformed state mimics can bypass selected upstream events and increase access to under-sampled conformations. Integrating these approaches may therefore provide a more complete series of structural snapshots than either strategy alone. Although demonstrated here for RSV polymerase, this framework may extend to other dynamic enzymes and macromolecular machines that traverse ligand-dependent conformational states during catalysis, translocation, assembly, or mechanical work. Combining reaction-derived trapping with state-matched assemblies, kinetic measurements, and time-resolved structural methods should help define more complete reaction landscapes and distinguish obligatory intermediates from accessible conformational states. For RSV and other NNS RNA viruses, further structural sampling of phosphodiester bond formation, pyrophosphate release, and different stages of elongation should clarify how dynamic polymerase architectures support RNA synthesis and may reveal transient conformations relevant to antiviral targeting.

## Data Availability

The cryo-EM density maps and atomic coordinates presented in the study are openly available in the Electron Microscopy Data Bank (EMDB; https://www.ebi.ac.uk/emdb/) and the RCSB Protein Data Bank (RCSB PDB; https://www.rcsb.org), respectively, with the following accession numbers: unreacted Tr3-mimic form A (EMD-77746, PDB 36PT) and unreacted Tr3-mimic form B (EMD-77747, PDB 36PU).

## Supplementary Materials

The following supporting information can be downloaded at https://www.mdpi.com/article/10.3390/microorganisms14081801/s1, Figure S1: Cryo-EM data processing of the RSV polymerase in complex with Tr3-mimic, resulting in two complex structures: unreacted Tr3-mimic forms A and B; Figure S2: The sequences, models, interacting residues, and cryo-EM maps of the RNAs in the unreacted Tr3-mimic forms A and B; Figure S3: Comparison of the RSV polymerase complex of unreacted Tr3-mimic forms A (this work, PDB: 36PT) and B (this work, PDB: 36PU); Table S1: Cryo-EM data collection, refinement, and validation statistics.

## Abbreviations

The following abbreviations are used in this manuscript:

RNA	Ribonucleic acid
NTP	Nucleoside triphosphate
ATP	Adenosine triphosphate
GTP	Guanosine triphosphate
RSV	Respiratory syncytial virus
Cryo-EM	Cryo-electron microscopy
NAC	Nucleotide addition cycle
NNS	Non-segmented, negative-sense
TrC	Trailer complementary
RMSD	Root-mean-square deviation
L	Large protein
P	phosphoprotein
RdRp	RNA-dependent RNA polymerase domain
Cap	Capping domain
CD	Connector domain
MT	Methyltransferase domain
CTD	C-terminal domain
NTD	N-terminal domain
OD	Oligomerization domain
