# Supplementary material for: Unreacted RSV Polymerase Structures Expand the Post-Translocation Landscape of the Nucleotide Addition Cycle
Source: Microorganisms. 2026 Aug 15;14(8):1801. doi: 10.3390/microorganisms14081801 (PMC13515464; doi:10.3390/microorganisms14081801)
Supplement: Supplementary file 1 [file microorganisms-14-01801-s001.zip › microorganisms-4457291-supplementary.pdf]

Supplementary Information

Unreacted RSV Polymerase Structures Expand the Post-Translocation Landscape of the Nucleotide Addition Cycle

Dongdong Cao, Eleanore Zhou, Jun Sim, Bo Liang*

Department of Biochemistry, Emory University School of Medicine, Atlanta, GA 30322, USA; dongdong.cao@emory.edu (D.C.)
*Correspondence: bo.liang@emory.edu

The PDF file includes:

Figures S1–S3
Table S1

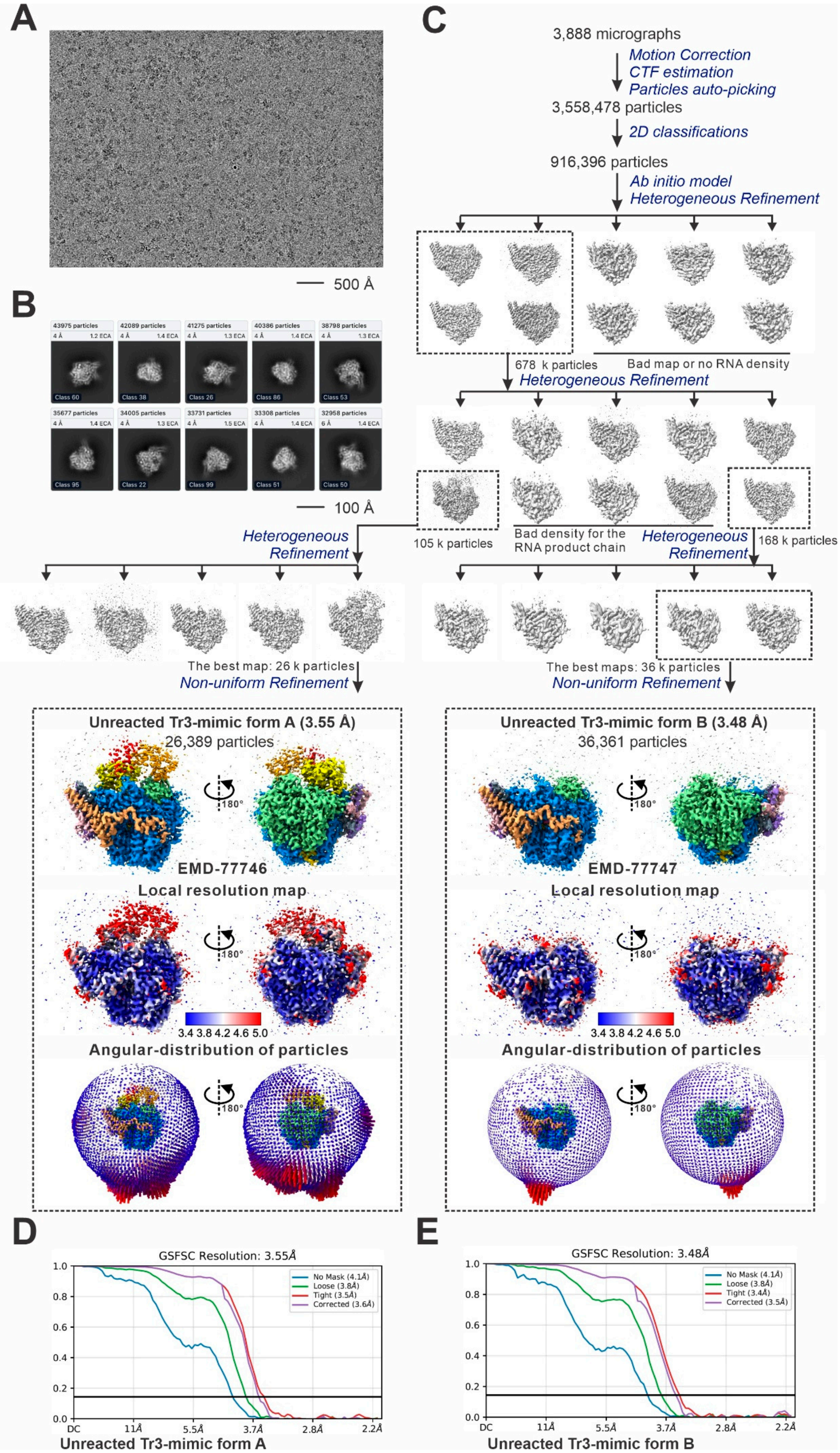

A
500 Å
B
100 Å
C
3,888 micrographs
Motion Correction
CTF estimation
Particles auto-picking
3,558,478 particles
2D classifications
916,396 particles
Ab initio model
Heterogeneous Refinement
678 k particles
Bad map or no RNA density
Heterogeneous Refinement
105 k particles
Bad density for the RNA product chain
Heterogeneous Refinement
168 k particles
Heterogeneous Refinement
The best map: 26 k particles
Non-uniform Refinement
The best maps: 36 k particles
Non-uniform Refinement
Unreacted Tr3-mimic form A (3.55 Å)
26,389 particles
EMD-77746
Local resolution map
3.4 3.8 4.2 4.6 5.0
Angular-distribution of particles
Unreacted Tr3-mimic form B (3.48 Å)
36,361 particles
EMD-77747
Local resolution map
3.4 3.8 4.2 4.6 5.0
Angular-distribution of particles
D
GSFSC Resolution: 3.55Å
No Mask (4.1Å)
Loose (3.8Å)
Tight (3.5Å)
Corrected (3.6Å)
Unreacted Tr3-mimic form A
E
GSFSC Resolution: 3.48Å
No Mask (4.1Å)
Loose (3.8Å)
Tight (3.4Å)
Corrected (3.5Å)
Unreacted Tr3-mimic form B

Figure S1. Cryo-EM data processing of the RSV polymerase in complex with Tr3-mimic, resulting in two complex structures: unreacted Tr3-mimic forms A and B.
(A) Representative micrograph of the RSV polymerase in complex with Tr3-mimic (5'-TrC12-4U-Tr3). Scale bar: 500 Å.

(B) Representative 2D class averages obtained from reference-free 2D classification. Scale bar: 100 Å.

(C) Datasets, classification, and refinement procedures using cryoSPARC. 3D Classes exhibiting clear densities for both the RNA template and product chains were pooled for final 3D classification. The final cryo-EM maps, local resolution maps, and the angular distribution of particles used for the final 3D reconstructions of unreacted Tr3-mimic forms A and B (both contoured at 0.20) are shown.

(D) Gold-standard FSC curves for unreacted Tr3-mimic form A (EMD-77746).

(E) Gold-standard FSC curves for unreacted Tr3-mimic form B (EMD-77747).

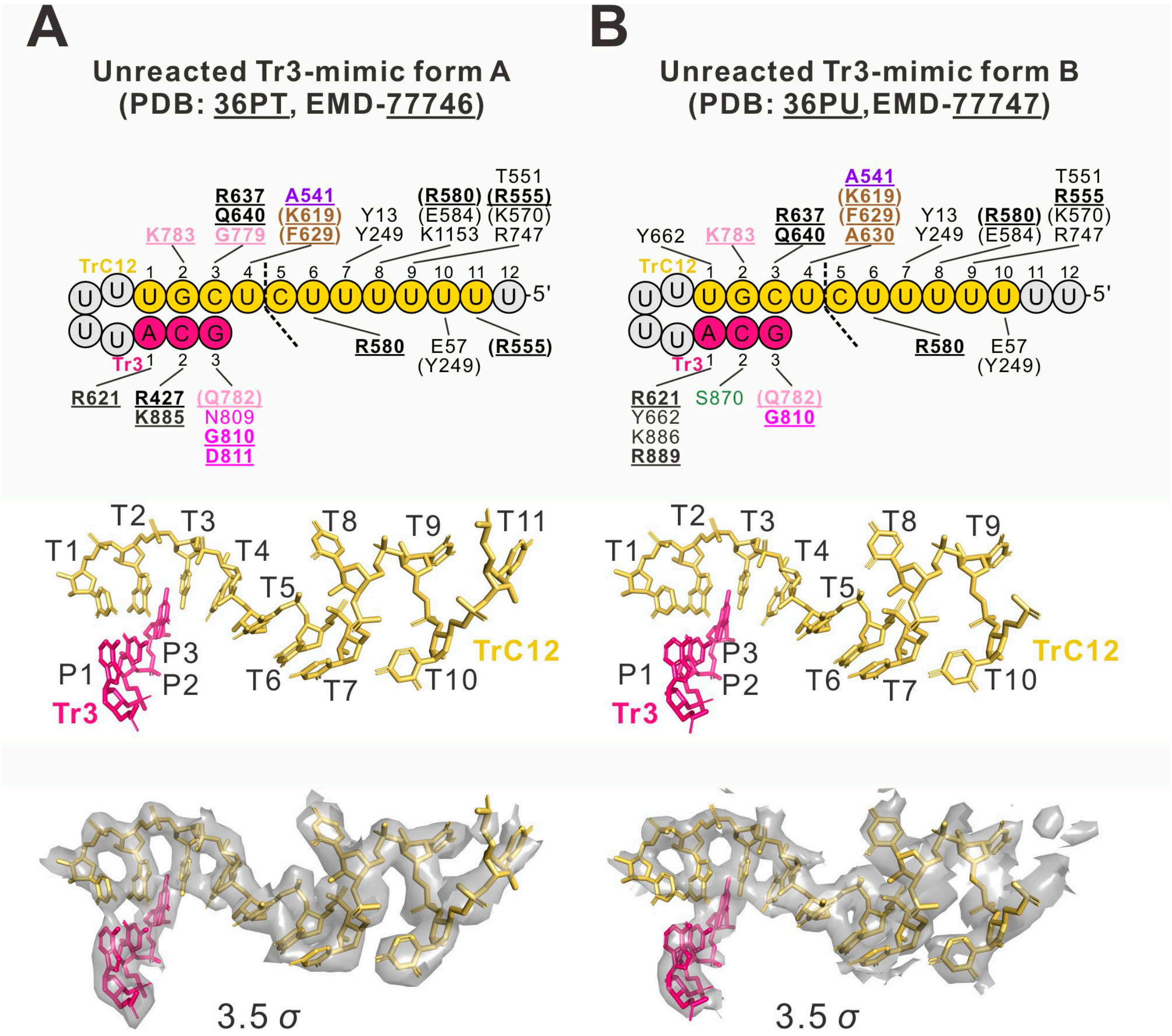

Figure S2. The sequences, models, interacting residues, and cryo-EM maps of the RNAs in the unreacted Tr3-mimic forms A and B.

The sequences, models, interacting residues, and cryo-EM maps of the RNAs in the RSV polymerase complexes of **(A)** unreacted Tr3-mimic form A and **(B)** unreacted Tr3-mimic form B. The contour level (in sigma units) is noted in each panel. The PDB ID and EMDB ID for each structure are underlined.

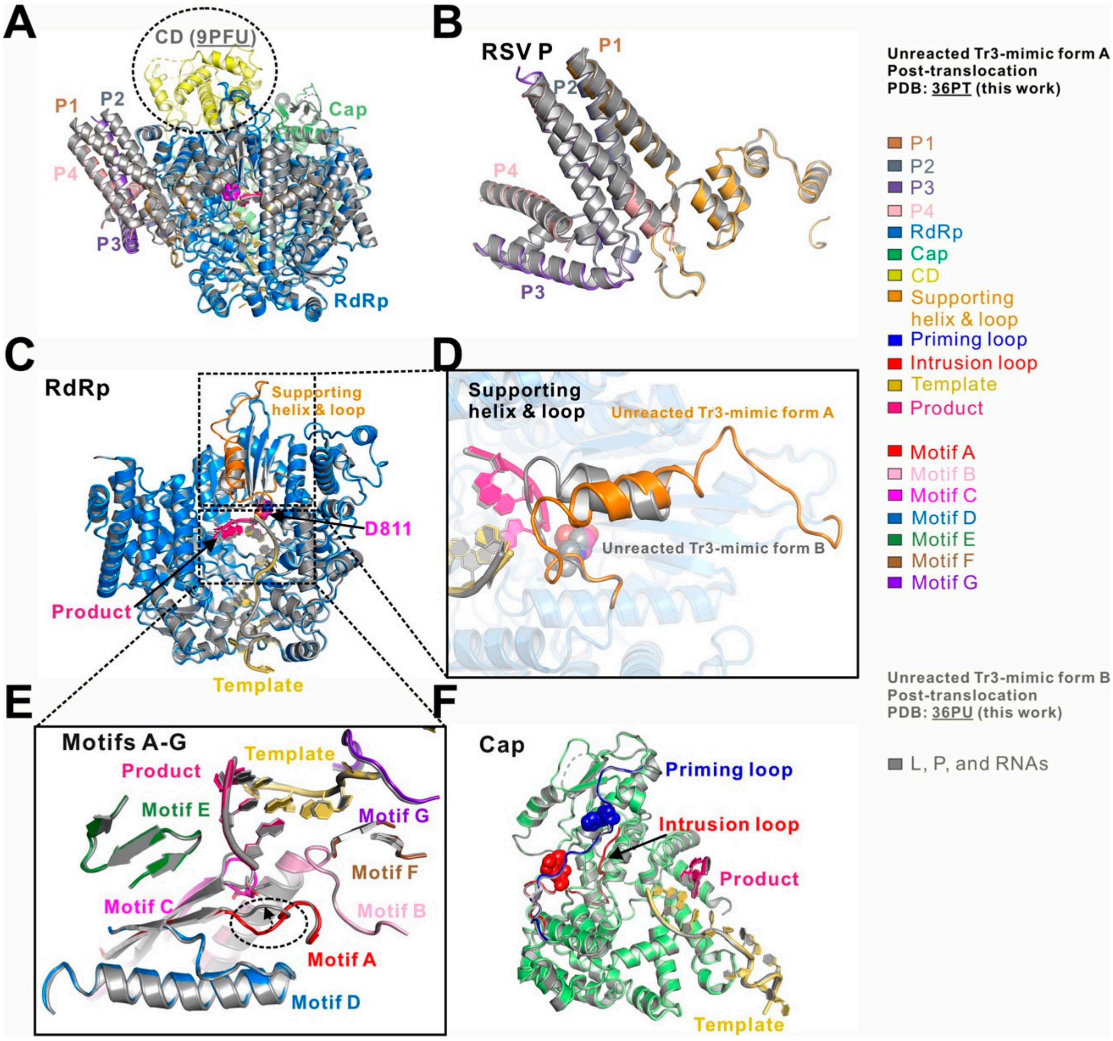

Figure S3. Comparison of the RSV polymerase complex of unreacted Tr3-mimic forms A (this work, PDB: 36PT) and B (this work, PDB: 36PU).

(A) The overall structural comparison between the unreacted Tr3-mimic forms A (PDB: 36PT) and B (PDB: 36PU, gray).

(B) The structural comparison of the RSV P proteins in these two structures.

(C) The structural comparison of the RdRp domains in these two structures.

(D) The structural comparison of the supporting helix and loops in the RdRp domains of these two structures.

(E) The structural comparison of the catalytic motifs A-G in these two structures.

(F) The structural comparison of the Cap domains in these two structures. The priming loop in the Cap domain of the unreacted Tr3-mimic form A is colored blue, and the intrusion loop is colored red.

The color of each domain or motif in the unreacted Tr3-mimic form A (PDB: 36PT) is indicated in the right part of the figure. The colors of motifs A to G apply only to panel E. The unreacted Tr3-mimic form B (PDB: 36PU) is shown as gray. The PDB ID is underlined.

Table S1. Cryo-EM data collection, refinement, and validation statistics

	**RSV polymerase+Tr3-mimic**	
**Data Collection and Processing**		
Magnification	81000	
Voltage (kV)	300	
Electron Exposure (e-/Å^2)	53.52	
Defocus range (μm)	-0.6 to -2.5	
Pixel size (Å)	1.058	
Symmetry imposed	C1	
Initial particle images (no.)	3,558,478	
**Final construct in structure**	Unreacted Tr3-mimic form A	Unreacted Tr3-mimic form B
**PDB code**	**36PT**	**36PU**
**EMDB code**	**EMD-77746**	**EMD-77747**
Final particle images (no.)	26,389	36,361
Map resolution (Å)	3.55	3.48
FSC threshold	0.143	0.143
**Refinement**		
Initial model used (PDB code)	9PFR	9PFR
Map sharpening *B* factor (Å^2)	-108.8	-100.8
Model composition		
Protein residues	1866	1659
Nucleotide	14	13
Ligands	0	0
Non-hydrogen atoms	15,451	13,686
B factors (Å^2)		
Protein	67.34	67.06
Nucleotide	64.63	81.89
Ligand	-	-
R.M.S. deviations		
Bond lengths (Å)	0.002	0.003
Bond angles (°)	0.564	0.565
Validation		
MolProbity score	1.60	1.59
Clashscore	6.32	7.34
Poor rotamers (%)	0.00	0.00
Ramachandran plot		
Favored (%)	96.30	96.88
Allowed (%)	3.70	3.12
Disallowed (%)	0.00	0.00
